# Supplementary figures and images for: Impaired hematopoiesis and embryonic lethality at midgestation of mice lacking both lipid transfer proteins VPS13A and VPS13C
Source: PLoS Biol. 2025 Sep 16;23(9):e3003393. doi: 10.1371/journal.pbio.3003393 (PMC12463328; doi:10.1371/journal.pbio.3003393)

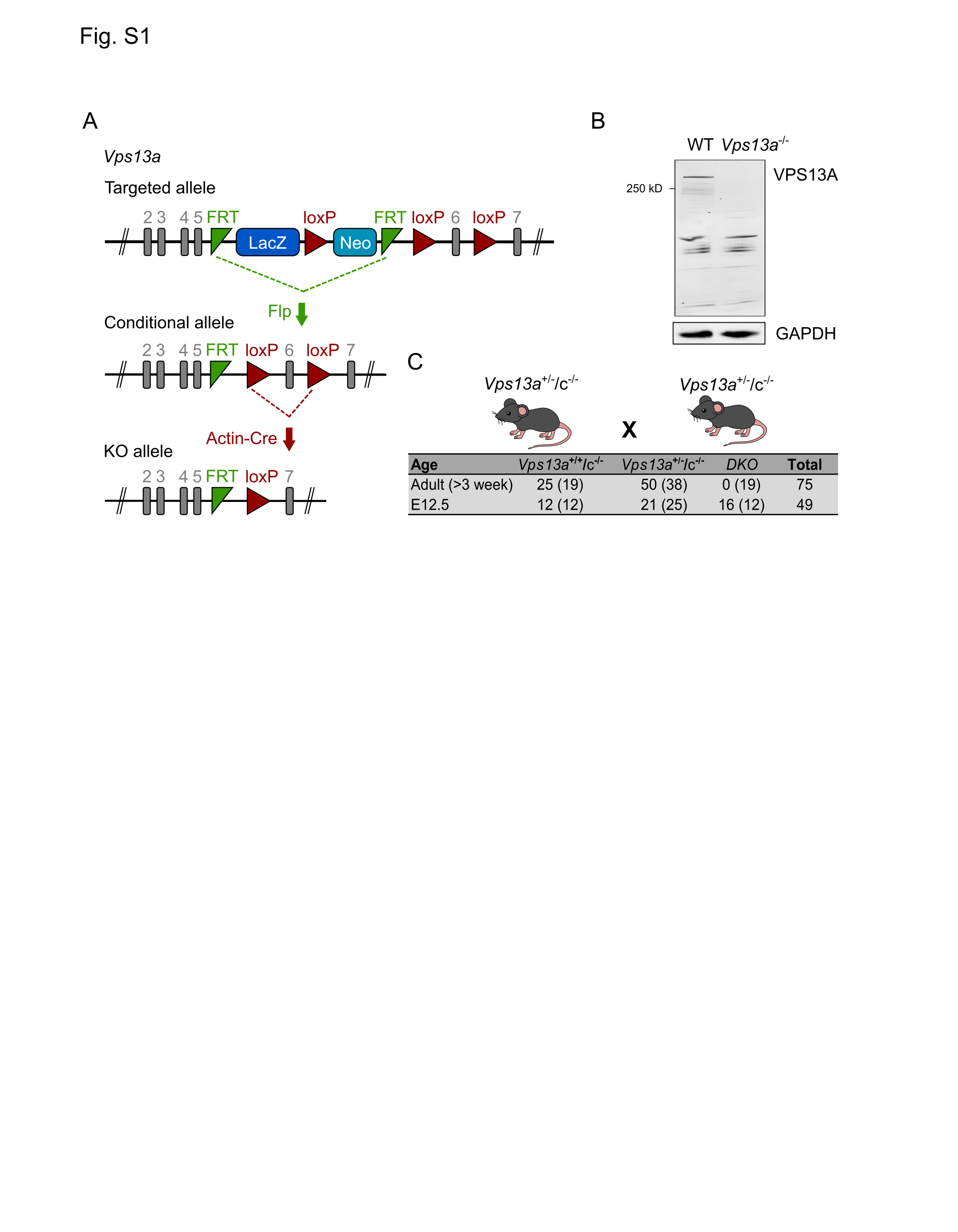

Supplement: S1 Fig — (A) Schematic overview of the process used for the generation of Vps13a KO mice to be mated to previously generated Vps13c KO mice. Vps13atm1a mice were first mated to Flp recombinase expressing mice to remove the LacZ and Neo cassettes to generate Vps13a conditional KO mice. Subsequently, mice were bred with β-actin-Cre expressing transgenic mice to remove exon 6 and generate constitutive full body Vps13a KO mice. (B) Anti-VPS13A western blots of lysates of cortical tissues from both WT and Vps13a KO mice confirming absence of VPS13A in the KO mice. (C) Genotyping results of adult mice and E12.5 embryos derived from Vps13a+/−/Vps13c−/− intercrosses. Values in the brackets indicate the number of expected mice/embryos based on mendelian distribution. (TIFF) [file pbio.3003393.s001.tiff]

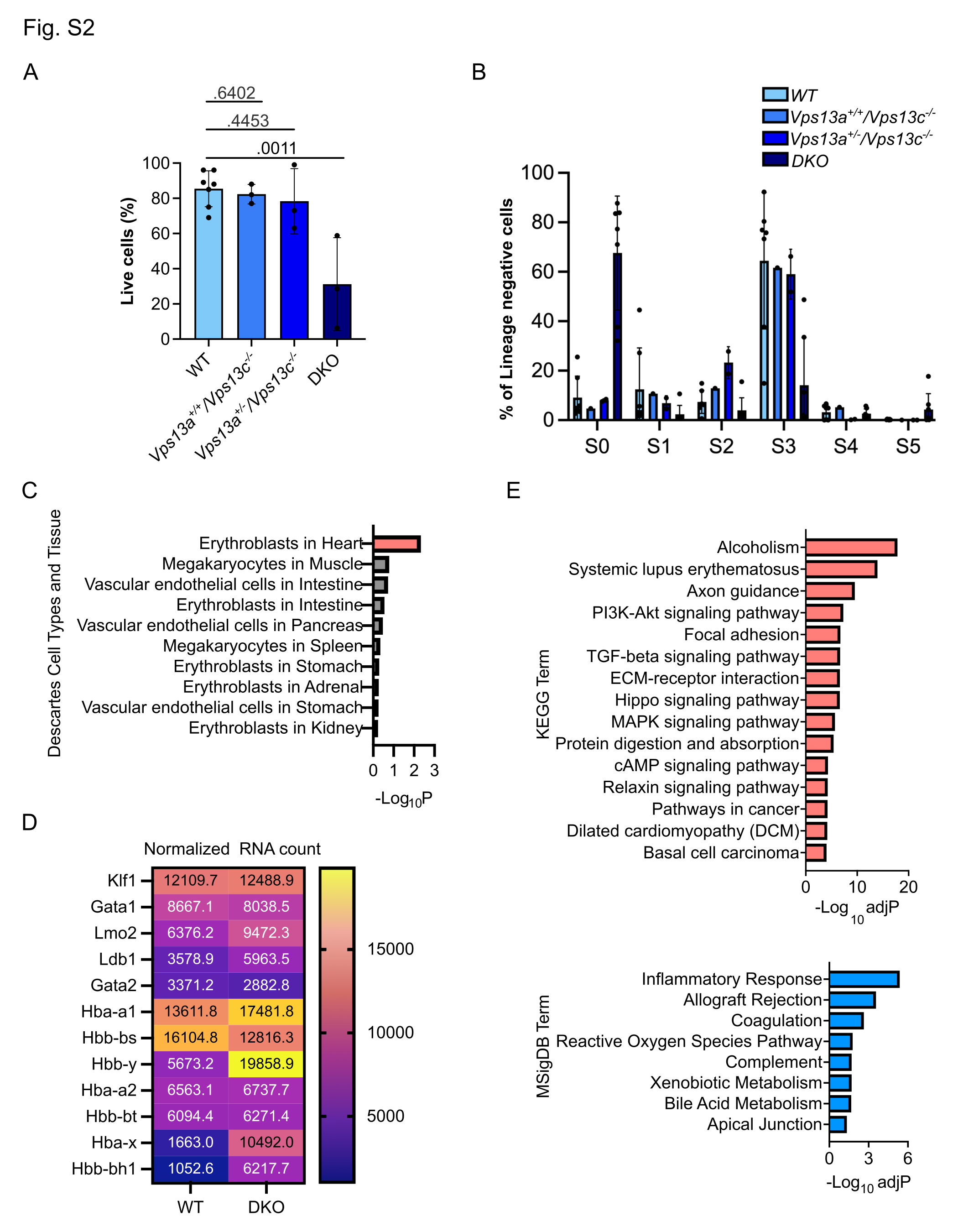

Supplement: S2 Fig — (A) Viability of cells derived from fetal livers assessed by trypan blue staining. Each dot represents one fetal liver with the specific genotype. (B) Percentage of each erythroid subset within the lineage negative gate. Each dot represents one fetal liver with the specific genotype. Results are presented as average plus SD of each subtype of erythroid cells. (C) The set of genes expressed by both WT and DKO S0 cells (p adj > 0.05 and |FC| < 1.2) was compared to the RNAseq profile of different cell types reported by the “Descartes Cell Types and Tissues Database”. The bar plot shows the top 10 cell types with transcriptomic profiles similar to those of S0 cells. (D) Heatmap showing normalized RNA count of key transcription factors that drives erythroid fate commitment and erythroid maturation in S0 cells. (E) Bar plot showing top 15 significantly upregulated (KEGG; top panel) and significantly downregulated (MSigDB; bottom panel) pathways based on adj P comparing DKO S0 cells versus WT. Raw data of this figure can be found at https://doi.org/10.5281/zenodo.15375803. (TIFF) [file pbio.3003393.s002.tiff]
