# Supplementary material for: Impaired hematopoiesis and embryonic lethality at midgestation of mice lacking both lipid transfer proteins VPS13A and VPS13C
Source: PLoS Biol. 2025 Sep 16;23(9):e3003393. doi: 10.1371/journal.pbio.3003393 (PMC12463328; doi:10.1371/journal.pbio.3003393)

Uncropped immunoblots

Fig. 4B

bands showed in the figure

bands used for quantification not showed in the figure

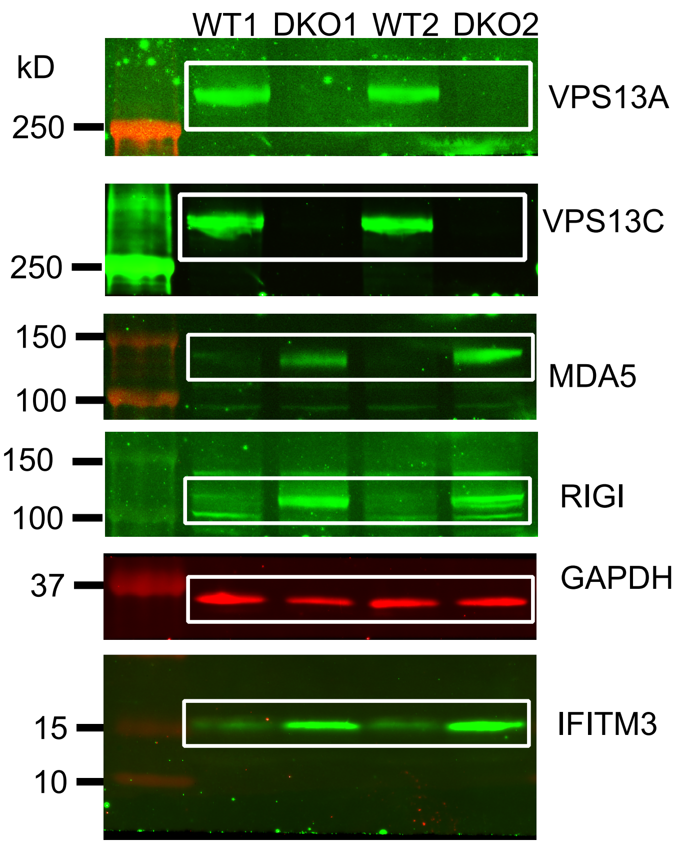

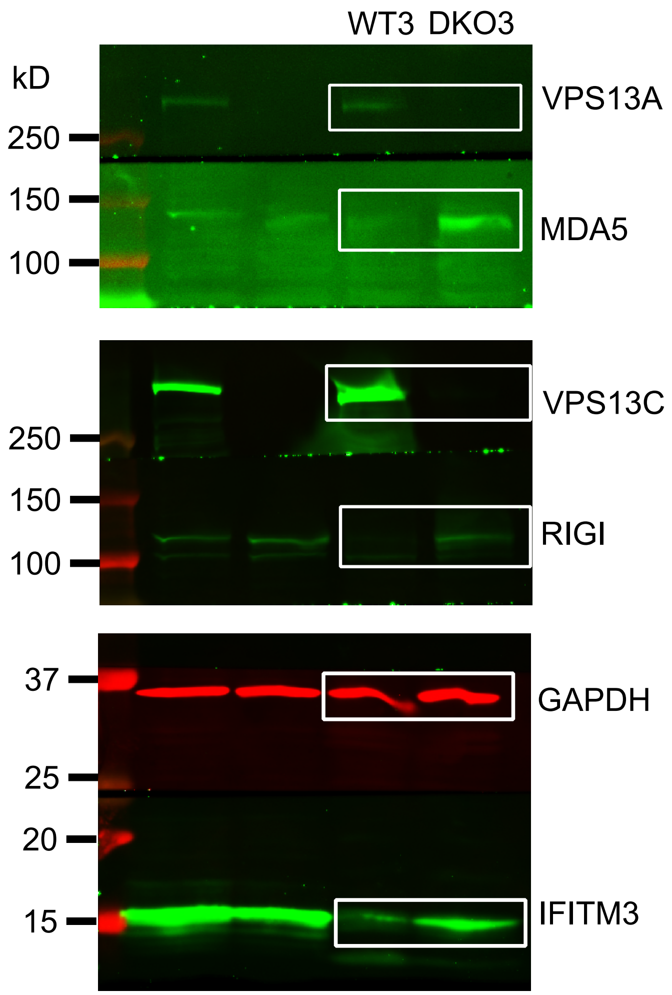

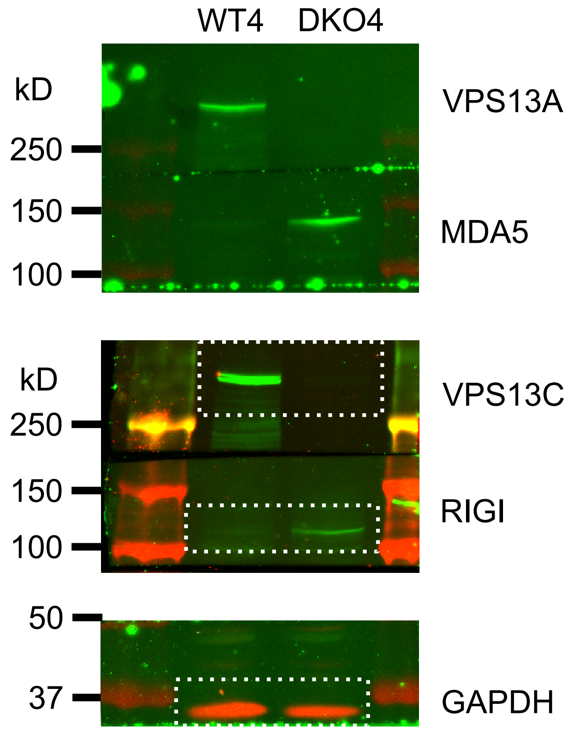

Uncropped immunoblots

Fig. 4C

bands showed in the figure

bands used for quantification not showed in the figure

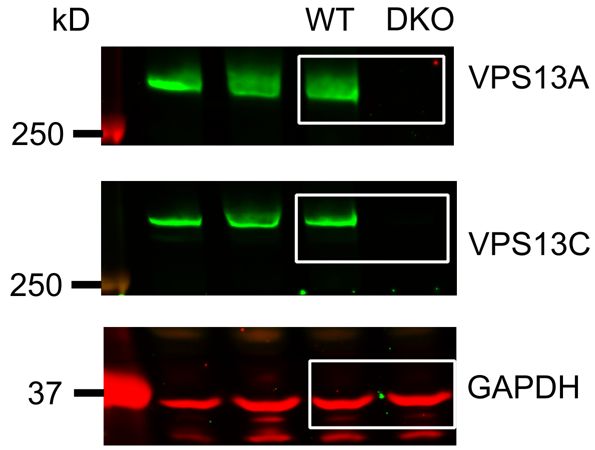

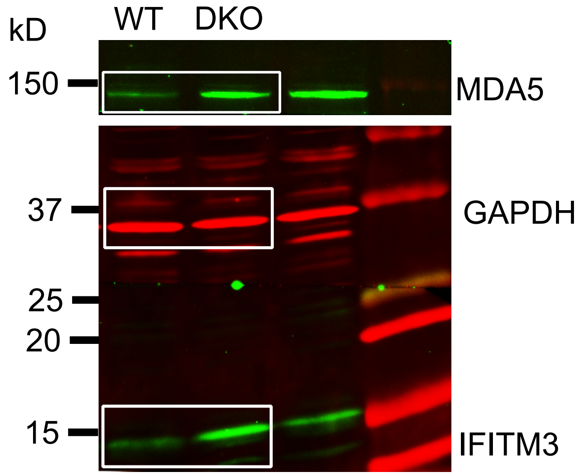

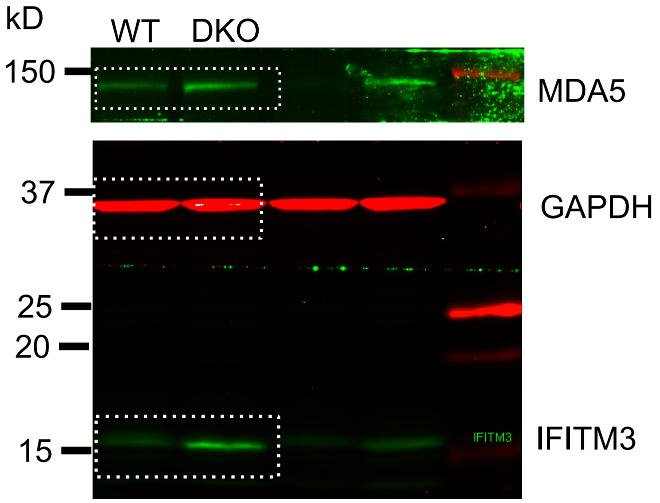

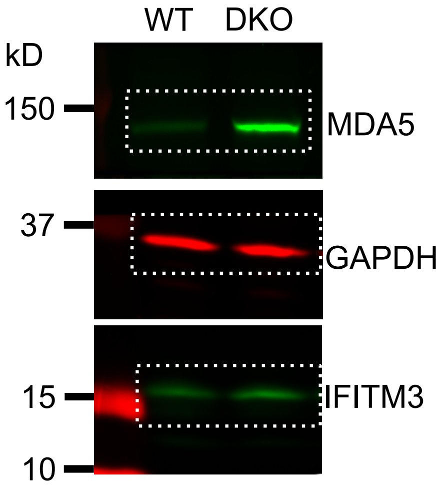

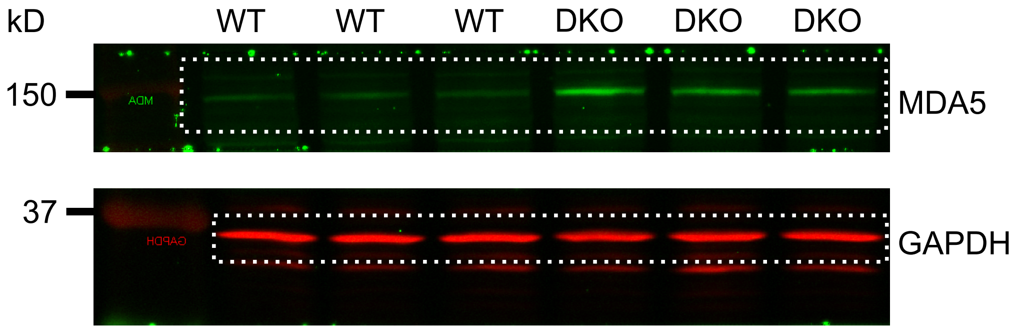

Uncropped immunoblots

Fig. 4D

bands showed in the figure

bands used for quantification not showed in the figure

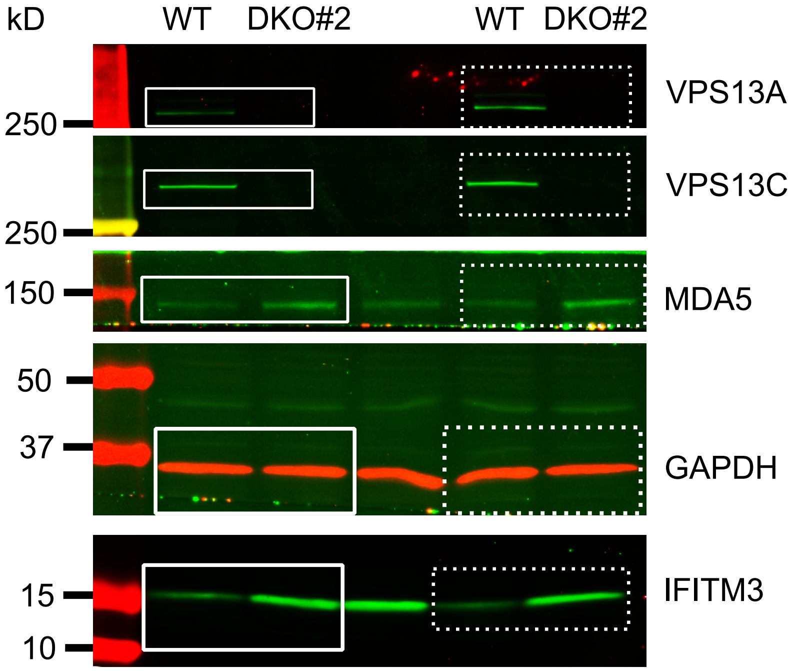

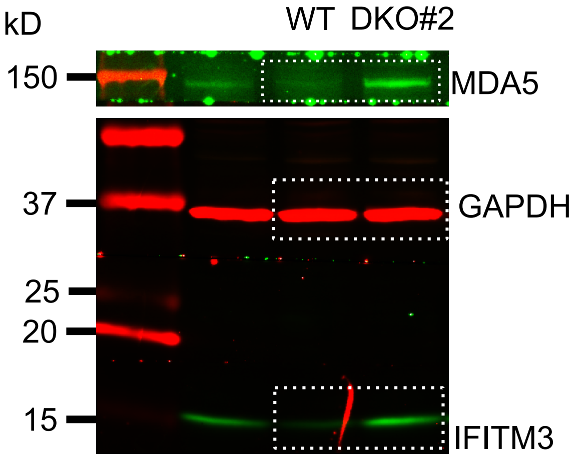

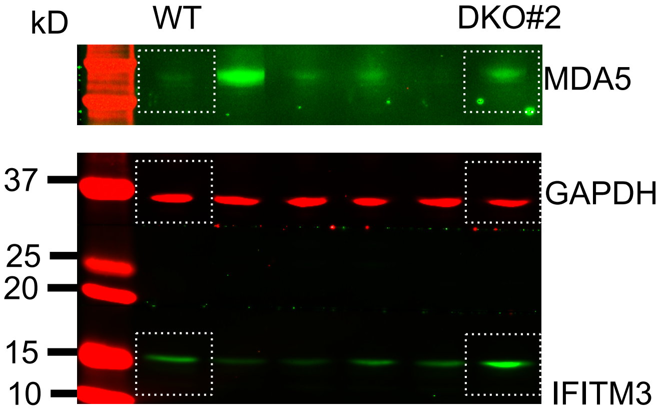

Uncropped immunoblots

Fig. 4E

bands showed in the figure

bands used for quantification not showed in the figure

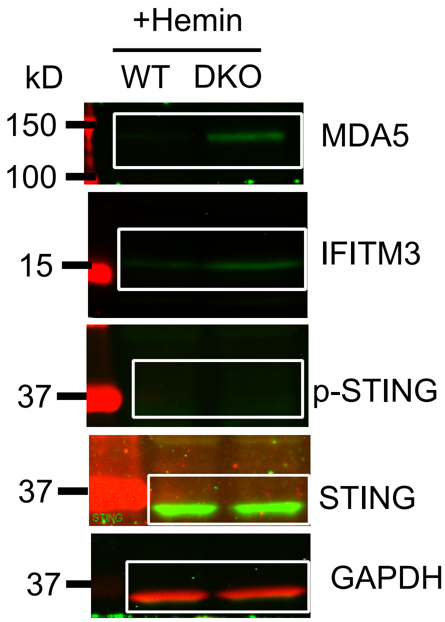

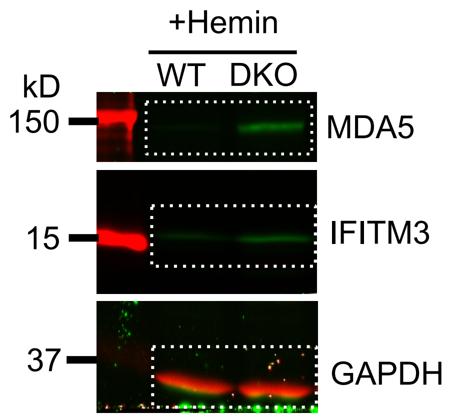

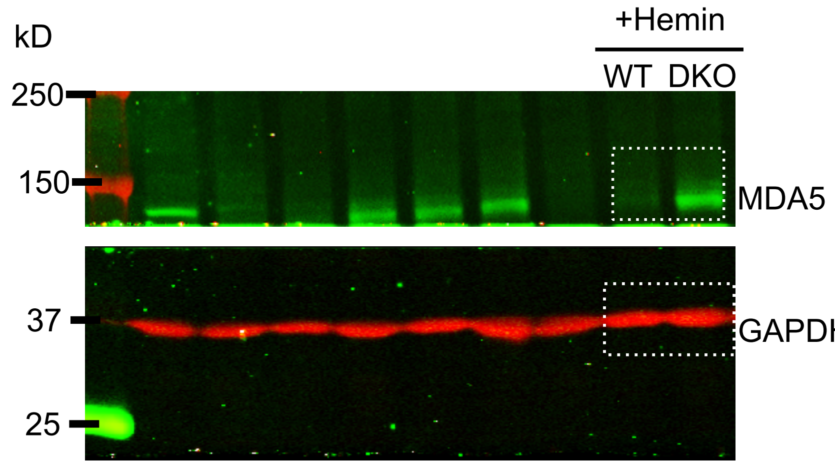

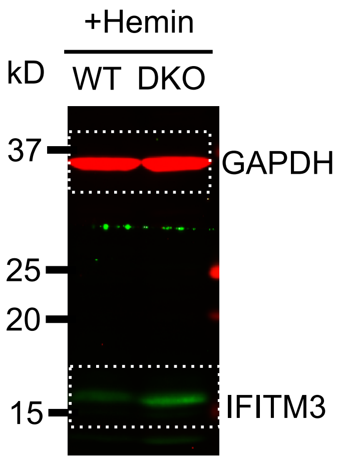

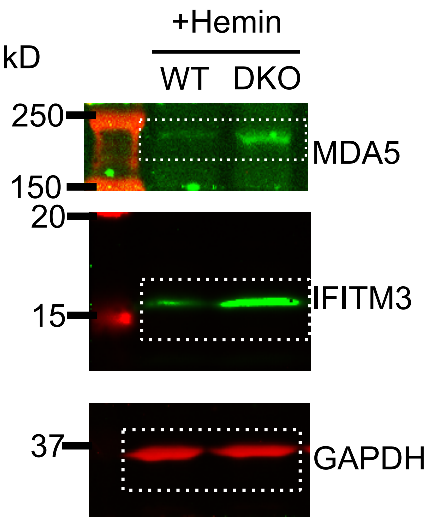

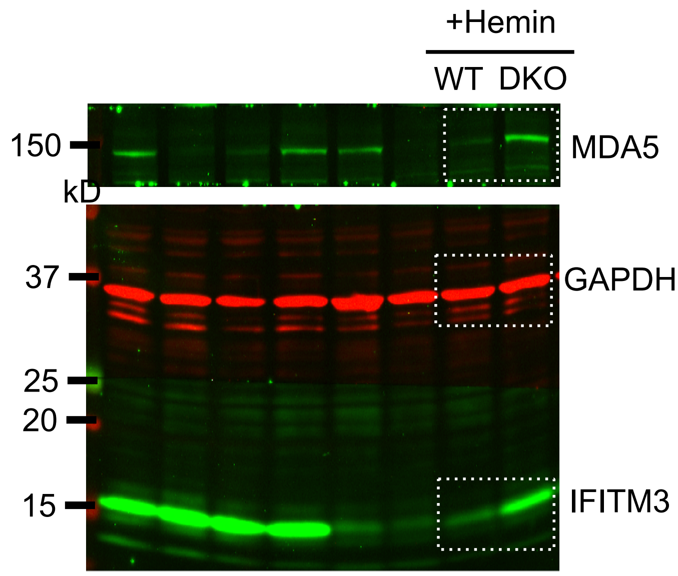

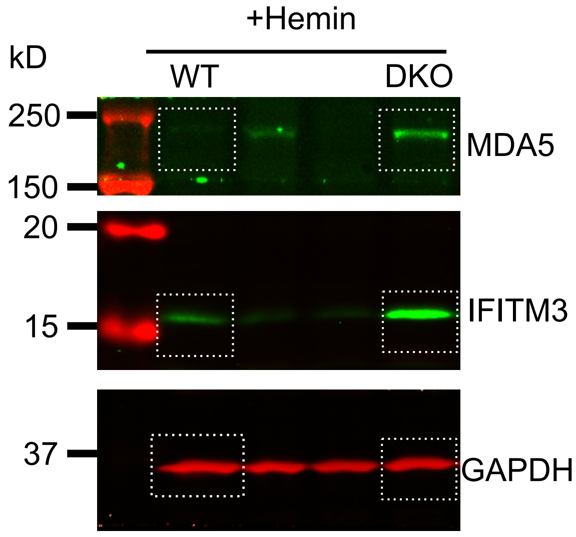

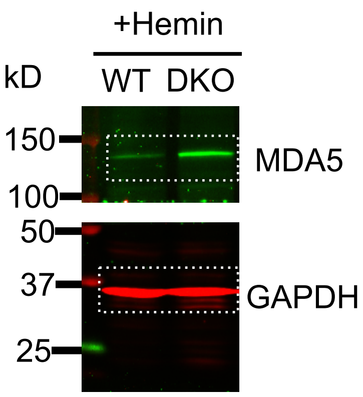

Uncropped immunoblots

Fig. S1B

bands showed in the figure

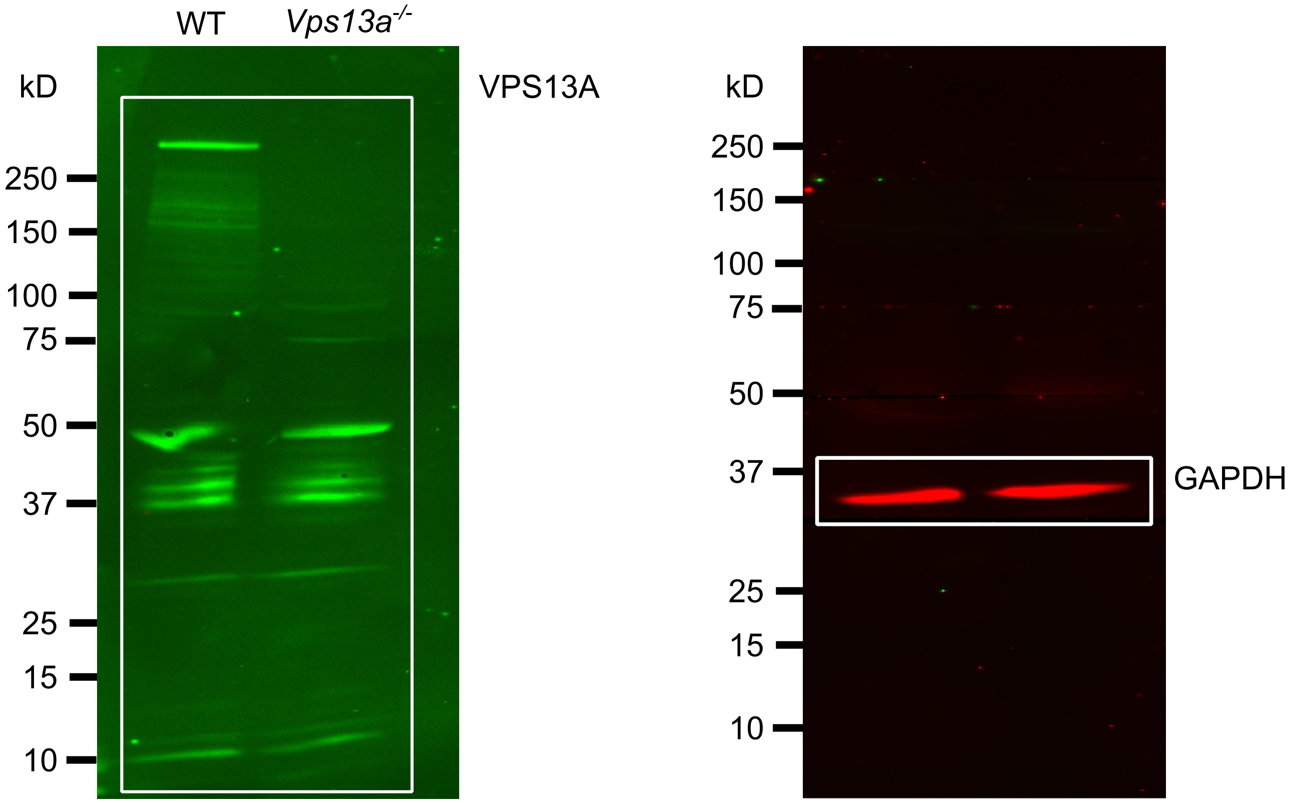

Supplement: S1 Raw Images — (PDF) [file pbio.3003393.s003.pdf]
