# Supplementary material for: Impaired hematopoiesis and embryonic lethality at midgestation of mice lacking both lipid transfer proteins VPS13A and VPS13C
Source: PLoS Biol. 2025 Sep 16;23(9):e3003393. doi: 10.1371/journal.pbio.3003393 (PMC12463328; doi:10.1371/journal.pbio.3003393)
Supplement: Attachment — Submitted filename: XU_Response_to_Reviewer_Comments_July_25_2025_auresp_3.docx [file pbio.3003393.s007.docx]

PBIOLOGY-D-25-01535R1

 Xu et al. (De Camilli)

"Defect in hematopoiesis and embryonic lethality at midgestation of Vps13a/Vps13c double knockout mice" 

Detailed rebuttal to the reviews

Reviewers’ text is in black and our response *in italic blue fonts*

*We thank the reviewers for their comments that helped us improve our manuscript.*

Reviewer #1:

In this paper the authors provide a brief report of data from double knockout, mouse embryos lacking the Vps13a and Vps13c genes and analyzed at a single timepoint (E12.5). They find that these embryos are small, pale, with a marked decrease in the size of the liver (figure 1A-E). Analysis of the fetal liver by flow cytometry with CD71 and Ter119 surface markers reveals a lack of normal erythroid differentiation with mutant cells almost entirely in the "S0" subpopulation (Figure 2). Comparison of RNA-Seq studies of normal and double knockout S0 cells revealed a down-regulation of genes associated with hematopoiesis and an up regulation of genes associated with interfere on signaling (Figure 3). The regulation of two of these genes (MDA5 and IFITM3) was also found in K562 cells lacking VPS13A and VPS13C (figure 4). In the discussion, the authors provide various speculations regarding the loss of there is lipid transfer genes and the regulation of genes associated with dsRNA
sensing.

Overall, the data are novel, logically laid out, and clearly presented.

*We thank this reviewer for her/his positive comment.*

However, there are a number of issues that bear consideration:

1. It is clear that the combined loss of Vps13a and Vps13c leads to embryonic lethality, however, it is not clear when that during embryogenesis they die. Did the E12.5 mouse have a heartbeat? (The timing of embryonic death may provide clues as to the etiology of the demise.)

*We have now mentioned that in initial exploratory studies we had found that double mutant embryos were consistently absent at E15, indicating their demise prior to that developmental stage. We apologize for not having included this information in the original manuscript. Similar pale embryos at E12.5 followed by embryos demise before E15 are also observed as a result of disruption of major erythropoiesis genes. We have now added to the discussion: “This phenotype and resulting embryonic death have been repeatedly observed in mice deficient in genes required for embryonic erythropoiesis [64, 65]”.*

*We did not observe a grossly evident heartbeat in E12.5 DKO embryos. However, we point out that embryos were removed shortly after euthanasia of the mother and that vascular structures, including heart, were less clearly visible in DKO embryos. It is likely that the combination of erythropoietic injury and embryonic death shortly after removal would have made a grossly evident heartbeat difficult to observe.*

2. The marked pallor at E12.5 indicates a severe defect in primitive erythropoiesis, since primitive red cells comprise nearly all of the circulating blood cells at this time point of mouse development. The flow cytometric analysis undertaken in the fetal liver indicates that definitive erythropoiesis is markedly blocked. Thus, the combined loss of these genes leads to profound defects in the differentiation of more than one lineage. This should be commented on and it may be useful to consider examining the circulating cells if there are enough to analyze in the mutant embryos.

*Although we focused on characterizing definitive erythropoietic defects in E12.5 DKO embryos, we agree that the pale color of DKO embryos also reveals a severe defect in primitive erythropoiesis, i.e the process that generates circulating primitive erythroid cells before E12.5. It is therefore likely that the double KO of Vps13a and Vps13c causes the impairment of both primitive and definitive erythropoiesis. We have now stated in the discussion: “Moreover, although in the characterization of the erythropoiesis defect, we focused on definitive erythropoiesis in E12.5 DKO embryos, the paler color of these embryos indicates a severe defect also in primitive erythropoiesis, which generates circulating primitive erythroid cells before E12.5. It is therefore likely that lack of both Vps13a/Vps13c causes the impairment of both primitive and definitive erythropoiesis”.*

*Concerning the question of whether it may be useful to consider examining the circulating cells, as the reviewer acknowledges, the small size of the DKO embryos makes studies of their circulating cells very challenging and we have not pursued this analysis.*

3. It is not clear that the S0 comparison is one of "apples to apples". It is assumed that they represent erythroid progenitors but that may not be the case since this population basically lacks all surface markers analyzed. Is there evidence from the RNA-Seq analysis that these cells are indeed erythroid progenitors?

*To isolate the S0 cell population, we followed the published protocol from the Sokolovsky’s lab (PMID: 20877475; PMID: 20877475). In this paper they have carefully characterized these cells and reported that the majority of S0 cells are erythroid precursors. Accordingly, we examined RNAseq data of our WT and DKO S0 cells to identify genes expressed by cells of both genotypes (padj>0.05 and |FC|<1.2) and compared this set of genes to the RNAseq profiles of different cell types (Descartes Cell Types and Tissues Database) finding that such set is the most similar to the RNAseq profile of erythroblasts (new Fig. S2C). Moreover, in both WT and DKO S0 cells, we observed high expression level of Klf1, Gata1, Gata2, Ldb1 and Lmo2, all key transcriptional regulators driving erythroid fate commitment and erythroid maturation, implying that these cells are indeed erythroid progenitors (new Fig. S2D). This new figure also shows that hemoglobin genes are highly expressed in WT and DKO S0 cells.*

*In order to incorporate these new data and figures we have partially rearranged the text under the previous subtitles: “Defect in erythrocyte differentiation” and “Activation of innate immunity in DKO erythroid progenitor cells” and combined this text under a new single subtitle: “Defect in erythrocyte differentiation and activation of innate immunity in DKO erythroid progenitor cells”*

4. While the focus of this paper is a characterization of erythropoiesis using flow cytometry and RNA-Seq approaches in the fetal liver, the embryonic sections in figure 1 reveal marked defects in the central nervous system. It is surprising that there is no analysis or even mention of the CNS, given that several of the authors are in Neuroscience departments and that humans/Drosophila with VPS deficiencies have neurodevelopmental issues.

*We wonder whether this comment may be due to the different macroscopic appearance of the developing brain in the sections shown. This difference, however, reflects the differences in the way brains have been cut, not anatomical differences.*

*We are definitely interested in the impact of VPS13A and VPS13C deficiency on the brain. However, the defects due to VPS13 A/C deficiency in drosophila and humans are age-related and not developmental defects. In humans they become manifest after more than 2 decades of life. In fact, given our interest in neuroscience, we are generating a conditional DKO mouse line to investigate neuronal defects in adult animals.*

5. In the Discussion, the authors speculate that a causal relationship between (potential) organelle defects and the activation of innate immunity. Some evidence that there are indeed organelle defects in blood cells would considerably strengthen this conjecture. For example, are there defects in mitochondria/lysosomes/Golgi/ER in the S0 cells?

*So far we do not have evidence of obvious changes in organelle shape or distribution in S0 cells. This is something we plan to explore in the future.*

Reviewer #2:

VPS13 proteins are a family of bridge-like lipid transport proteins (BLTPs) that mediate bulk lipid transfer at distinct subcellular contact sites. Among them, VPS13A and VPS13C are the most closely related and specific to vertebrates. In humans, loss-of-function mutations in either gene lead to distinct late-onset neurodegenerative diseases. However, their roles during early development have remained largely unclear. This study shows that while mice lacking either VPS13A or VPS13C individually are viable, double knockout (DKO) embryos die at midgestation. The authors identify impaired hematopoiesis and activation of innate immunity—specifically the RIG-I-mediated interferon response—as possible contributors to this lethality. Overall, the study provides valuable insights into the previously unrecognized functions of VPS13 in early mammalian development.

*We thank this reviewer for his/her positive comment about our work.*

The main criticism is that the cause of lethality is not established. It would require tissue-specific KO or tissue specific rescue to ascertain that liver hematopoietic defects are causal and cell-autonomous to the lethality, which is beyond the scope of the present study. Since the hematopoiesis defect is presented with appropriately careful wording, this is not an issue, but perhaps the authors may want to comment on the fact that even if liver development is strongly affected, it does not mean that the activity of VSP13A and C is in fact required in the liver.

*We have made changes to the text to address this suggestion as shown below.*

*In Results we state: “Consistent with a defect in erythropoiesis, fetal livers of E12.5 DKO embryos were smaller and paler relative to those of control littermate embryos (Fig. 1C, and D).”*

*In the Discussion we state: “While a major phenotype observed the DKO embryos is a small liver, such defect is likely explained by defective erythropoiesis, as cells of the erythroblastic lineage are by far the most important contributor to the liver mass at this embryonic stage [63]”.*

Similarly, the discussion is focused on the topic of redundancy. It is not clear from the data whether both proteins are indeed redundant, or whether the absence of either cause a mild stress, and the absence of both cause an additive and lethal one (with both stresses being more or less independent). That the group has localized both proteins to different contact sites might argue for the latter. Yet, given the mystery that shrouds the localization mechanisms for both proteins, it is also conceivable that, outside of highly transformed cultured cells, where the localization experiments have been done, the localization pattern of both proteins is much more overlapping than previously thought, consistent with a redundancy phenomenon. At present the discussion offers a very short paragraph (the last one) to discuss these possibilities; a much larger one is dedicated to the cause of the lethality (erythropoiesis, cGAS-STING pathway), which, in the absence of tissue-specific KO and rescue, remains speculative.

*We have added the following sentences to the end of the discussion:*

*“It also remains possible that VPS13A and VPS13C have yet uncharacterized overlapping subcellular localizations (for example a localization of VPS13C at ER-mitochondria contacts, where VPS13A is also present, had been suggested [10, 24]). Moreover, both proteins have been found at ER-lipid droplets contacts [10, 16], although the functional significance of this localization remains unclear. So far, localization studies of these two proteins have relied primarily on their overexpression (tagged proteins) in cell lines, given the limitations of currently available antibodies. Future studies of endogenously tagged proteins and in different tissues will be needed to obtain a full picture of the cellular and subcellular distributions of these proteins under control conditions and in different functional states towards a better understanding of their roles in physiology and disease.”*

Reviewer #3:

This is an extremely well-conducted and clearly reported study that addresses the role of VPS13A and VPS13C in erythropoiesis.

The use of a double knockout (DKO) model convincingly demonstrates the functional redundancy of these lipid transport proteins and provides robust in vivo evidence for their critical involvement in red blood cell development and the regulation of innate immune responses. The authors support their conclusions with an extensive array of molecular, cellular, and hematological assays, offering a comprehensive characterization of the erythropoietic phenotype.

The manuscript is highly relevant and contributes important new insights into the connection between intracellular lipid transport, organelle homeostasis, and hematopoietic differentiation. Particularly noteworthy is the observation that disruption of inter-organelle lipid transport leads to erythroid-specific defects alongside aberrant activation of innate immune pathways.

*We thank this reviewer for these most positive comments.*

However, one aspect remains insufficiently explored: the mechanistic link between VPS13A/C deficiency and the pronounced activation of interferon signaling specifically in erythroid cells. While the authors convincingly demonstrate induction of interferon-stimulated genes (ISGs) and involvement of RIG-I/MDA5 and cGAS-STING pathways, the upstream molecular events leading to this activation are not adequately discussed or experimentally addressed.
A plausible scenario worth further consideration involves the role of VPS13A and VPS13C in autophagy and mitophagy. These processes are essential for proper erythroid maturation, particularly in the clearance of mitochondria. Disruption of autophagy could lead to the accumulation of damaged mitochondria and leakage of mitochondrial DNA or double-stranded RNA into the cytosol—recognized triggers of cGAS-STING and RIG-I/MDA5 signaling, respectively (West et al., 2015; White et al., 2014; Sliter et al., 2018). VPS13A has been implicated in autophagy regulation, while VPS13C has a documented role in lysosomal lipid homeostasis. Defective mitophagic flux in erythroid precursors may therefore underlie the immune activation phenotype observed in this model.
Assessing whether the combined loss of VPS13A/C alters autophagy or mitophagy flux would substantially strengthen the study and offer a mechanistic explanation for the observed interferon response in erythroid cells.
In conclusion, this study represents a significant advancement in the field and opens new avenues for future research. Including or more fully discussing the potential link between autophagy dysregulation and innate immune activation would deepen the mechanistic insight and enhance the impact of the work.

*Thank you for this suggestion. In response to this comment we made the following additions:*

*Abstract: we added the words in bold: Activation of innate immunity may result from loss of integrity of the membranes of intracellular organelles, such as mitochondria and autophagic lysosomes,* ***or to impaired autophagy,*** *due to the absence of these lipid transport proteins.*

*End of the Introduction: we slightly modified the last sentence which originally read “We speculate that activation of innate immunity may result from loss of the integrity of the membranes of intracellular organelles leading to leakage of mitochondrial material from mitochondria or autophagic lysosomes, and that erythroid ….” and now reads “We speculate that activation of innate immunity may result from loss of the integrity of the membranes of intracellular organelles* ***or to impaired autophagy****, and that erythroid ….”*

*Discussion: We have added this text to the end of the Discussion “It is also possible that a primary defect in autophagy may be responsible for, or contribute to, the activation of innate immunity in erythrocytes precursors lacking both VPS13A and VPS13C, as roles of VPS13 family proteins in autophagy have been reported [33, 67-70]. For example, the single yeast Vps13 protein cooperates with Atg2 (a bridge-like lipid transfer protein of the VPS13 protein family) in the growth of the autophagic membrane [67-69] and both mammalian VPS13A and VPS13C were proposed to participate in this process via an interaction with the scramblase ATG9, a well-known interaction partner of ATG2 [71]. Moreover, VPS13A was implicated in autophagy by studies of human and mouse skeletal muscle [33]. Erythrocytes precursors may be especially sensitive to a defect in autophagy, as their maturation involves the autophagic clearing of intracellular organelles, including mitochondria [72-74]. A defect in this process would prevent appropriate sequestration of mitochondrial nucleic acids and other toxic material resulting in activation of innate immunity[75]. Accordingly, in an unbiased proteomics screen for elevated proteins in autophagy deficient cells, the RIG-I signaling pathway was one of the top hits [76] and the RIG-I pathway was enhanced in cells deficient in the ATG5-ATG12 complex, which is required for autophagy [77, 78]”.*
